# RNA-Binding Proteins in mRNA Splicing: Orchestration of Altered Phosphorylation of RNA-Binding Domains via Kinase Regulatory Networks

**DOI:** 10.3390/ijms27219965

**Published:** 2026-11-06

**Authors:** Ashika Bangera, Samseera Ummar, Alimath Sambreena, Athira Perunelly Gopalakrishnan, Poornima Ramesh, Rajesh Raju

**Affiliations:** Centre for Integrative Omics Data Science (CIODS), Yenepoya (Deemed to be University), Mangalore 575018, India; bangera.ashika18@gmail.com (A.B.); samshisam87@gmail.com (S.U.); sambreenaibrahim75@gmail.com (A.S.); ammuperunelly@gmail.com (A.P.G.)

**Keywords:** mRNA splicing, RNA-binding domain, phosphorylation, kinase, RNA processing, mass spectrometry, regulatory networks

## Abstract

RNA-binding proteins (RBPs) are crucial regulators of post-transcriptional gene expression, orchestrating essential processes including splicing, mRNA transport, translation modulation, and mRNA decay. Phosphorylation of RBPs is crucial for regulatory mechanisms, mediating how extracellular stimuli control intracellular signaling and cellular decisions. In this study, mass spectrometry-based phosphoproteomic data were systematically integrated and harmonized, and Fisher’s exact test (*p*-Value < 0.05) was applied to quantify conserved co-differential phosphorylation across pairs of phosphorylation sites in comparison to a chosen set of RNA-binding proteins (HNRNPM, SLU7, and SRSF5). For each such pair, data were benchmarked across >800 phosphorylation profiling and 200 differential phosphorylation datasets, identifying predominant co-differential phosphorylation patterns that accounted for >75% of all observed phosphorylation events. Major phosphorylation hotspots were identified in HNRNPM, SLU7, and SRSF5 RNA-binding proteins. High-confidence pairs were further refined using secondary filters, yielding a focused set of functionally relevant relationships in mRNA and alternative splicing. As the RNA-binding activity is mediated by RNA-binding proteins (RBDs), co-differentially regulated phosphorylation sites were mapped to RBDs, and upstream kinases modulating such phosphorylation were derived from experimental, semi-experimental and predictive sources. Overall analysis confirmed phosphorylation of RBPs on their RBDs through the upstream kinases, modulating mRNA and alternative splicing through a conserved network of co-differential phosphorylation events. With further validation, the data imply a platform for identifying relevant targets and markers for studying mRNA splicing and RNA-binding activity, central to RNA processing.

## 1. Introduction

Splicing is an essential post-transcriptional modification process that transforms pre-mRNA/precursor transcripts into mature mRNAs [1]. Splicing is an important coordinator of mRNA export, stability, translation and isoform expression through alternative splicing, resulting in transcriptome diversity, genome stability and cellular identity [2]. Splicing is facilitated by RNA-binding proteins (RBPs), which directly and indirectly mediate several key regulatory stages of the process [3,4,5]. RBPs are a large and essential class of regulatory proteins involved in RNA splicing, processing, transport, and stability. Dysfunction of splicing factors, including RBPs, and their post-translational modifications contributes to oncogenesis, apoptosis dysregulation, and metabolic dysfunction, resulting in various disorders [6,7]. Human proteins contain nearly 2000 RBPs, most of which function through specialized RNA-binding domains (RBDs) that interact with specific mRNA regions. Key RBDs include the RNA recognition motif (RRM), K-homology domain (KH), RGG (Arg-Gly-Gly) box, zinc finger, double-stranded RBDs (dsRBD), Pumilio/PUF domain and Piwi/Argonaute/Zwille (PAZ) domain [8]. Among these, the RRM is the most prevalent and functionally significant domain, playing critical roles in RNA metabolism and post-transcriptional gene regulation [9].

Mass spectrometry-based phosphoproteomics is a powerful platform for studying global changes in protein flux and phosphorylation events significant in signal transduction. Protein phosphorylation is a crucial post-translational modification, as it governs the regulation of various cellular processes [10]. Phosphorylation of RBPs on their RRM and KH domains are known to control RNA binding, protein–protein interactions, and mRNA decay in response to extracellular cues and cell cycle checkpoints, with serine phosphorylation on these domains implicated in cancer progression [11]. Additionally, phosphorylation of RRM and KH domains can induce alterations in the structure, subcellular localization, and protein interactions, thereby impacting cancer development [12]. With the intent of studying the phosphorylation landscape of RBDs across RBPs, we prioritized three pivotal RBPs: Heterogeneous Nuclear Ribonucleoprotein M (HNRNPM), Pre-mRNA-splicing factor (SLU7), and Serine/Arginine-Rich Splicing Factor 5 (SRSF5). These proteins were prioritized due to their well-characterized involvement in splicing regulation and disease pathobiology, and the availability of abundant phosphoproteomic datasets that quantify phosphorylation of these proteins.

HNRNPM is a member of the heterogeneous nuclear ribonucleoprotein (hnRNP) family, which is predominantly localized in the nucleus and has also been found to be associated with chromatin [13], where it participates in pre-mRNA processing, splicing regulation, mRNA export, stability, and translation, largely through RRMs that mediate alternative splicing regulation [14]. It is known to be phosphorylated at S574 in a p38-MAPK-dependent fashion [15,16]. In mouse models, phosphorylation of HNRNPM at several serine residues (S85, S431, S480, S574, and S636) regulated by upstream signaling through Toll-like receptor 2 (TLR2) activation during *Mycobacterium tuberculosis* infection has been reported [15,17]. In humans, such functional aspects related to RBDs are yet to be completely illustrated. Similarly, SLU7 is an evolutionarily conserved mRNA-binding protein that functions as an essential splicing factor ensuring the correct selection of 3′ splice sites [18,19]. The SLU7 protein contains several conserved functional domains, including a nuclear localization signal (NLS), a zinc knuckle motif, four leucine-rich repeat regions, and a lysine-rich domain [20]. SLU7 predominantly localizes to the nucleus in a speckled distribution; however, upon stress stimuli such as UV-C irradiation or heat shock, it relocalizes to the cytoplasm via activation of the JNK and p38 MAPK pathways [20]. SLU7 is a multifunctional protein essential for the second step of pre-mRNA splicing, catalyzing exon ligation to ensure accurate mRNA maturation [21]. Beyond splicing, SLU7 is implicated in the regulation of DNA methylation, transcriptional control, chromosomal stability, and mitotic progression [22]. Furthermore, loss of SLU7 induces hepatocellular proliferation and triggers a shift toward a tumor-like glycolytic phenotype [23]. SRSF5, also known as SRp40, belongs to the serine/arginine-rich splicing factor (SRSF) family, which regulates splice site recognition and spliceosome assembly during precursor mRNA splicing [24]. SRSF5 is primarily localized to nuclear speckles and can also be found in the nucleoplasm. Structurally, SRSF5 exhibits a classical structure of the SR protein, including one RNA recognition motif (RRM) domain, an RRM homology domain, and a serine and arginine (RS) domain. SRSF5 controls mRNA splicing, including exon inclusion and skipping, mRNA export, and mRNA stability [25]. The insulin-activated PI3K-AKT pathway activates SRSF5 via AKT2-mediated phosphorylation, which promotes PKCβII exon inclusion and the production of mature PKCβII mRNA, increasing glucose transport activity, and ensuring skeletal muscle energy metabolism [26].

Although the functional roles of HNRNPM, SLU7, and SRSF5 have been extensively characterized, their phosphorylation aspects remain largely unexplored from a conserved co-differential regulation point of view. Therefore, we hypothesize that co-differential regulation protein networks governing RNA-binding function show conserved phosphorylation patterns, common upstream kinases and interactions relevant to RNA-binding activity. Hence, we systematically identified the predominant phosphorylation sites within these three proteins and further investigated conserved co-differential regulation of RBD-containing RBPs with HNRNPM, SLU7, and SRSF5. We specifically mapped potential kinases that modulate the phosphorylation of RBDs of the RBPs showing conserved co-differential regulation with the proteins of interest to derive co-differential regulatory networks relevant in splicing. Further, we mapped the kinase that phosphorylates the RBDs on co-differentially regulating RBPs. Although some of these findings need to be tested in experimental settings, we believe these findings lay a valuable foundation for future studies exploring the therapeutic potential of targeting phosphorylation-mediated regulation in RBPs with consequences in splicing.

## 2. Results

### 2.1. Assembly of Global Phosphoproteomic Data

We systematically curated and assembled 3825 publicly available phosphoproteomic datasets from PubMed-indexed articles that specifically focused on human cell lines. We further filtered and obtained 786 profile and 219 differential datasets for HNRNPM; 857 profile and 127 differential datasets for SLU7; and 615 profile and 134 differential datasets for SRSF5. These datasets exclusively include high-confidence phosphorylation sites and respective phosphopeptide quantities (see details in the Section 4). An in-house mapping tool was employed for comprehensive annotation of genes and phosphorylation sites to their corresponding UniProt accessions (Supplementary Tables S1–S6).

### 2.2. Major Phosphorylation Sites Observed in Mass Spectrometry-Based Proteomics

The predominant phosphorylation sites in HNRNPM, SLU7, and SRSF5 were identified by ranking high-confidence phosphorylation sites by their frequency of detection across various biological and experimental conditions in the profile and differential datasets. Among the 52 phosphorylation sites in the profile datasets of HNRNPM, the phosphorylation site S633 was detected in most experimental and biological conditions with a frequency of 464 (Figure 1A). Similarly, the profile dataset of SLU7 includes a total of 20 phosphorylation sites, among which S215 appeared with the highest frequency of 831 (Figure 1B). The profile dataset of SRSF5 had a total of 28 phosphorylation sites, out of which S233 appeared with the highest frequency of 627 (Figure 1C). Differential datasets of HNRNPM showed differential abundance of 27 phosphorylation sites, out of which the predominant phosphorylation sites S528, S618, S633, and S701 showed a frequency of 73, 70, 55, and 51, respectively (Figure 1A). Differential datasets of SLU7 showed differential abundance of 11 phosphorylation sites, out of which the predominant phosphorylation sites S215, S515 and S235 showed frequencies of 82, 33, and 29, respectively (Figure 1B). Similarly, differential datasets of SRSF5 showed differential abundance of 15 phosphorylation sites, out of which the predominant phosphorylation sites S233, S229, S250 and S231 showed frequencies of 79, 63, 54, and 52, respectively (Figure 1C).

Interestingly, HNRNPM (S701) and SLU7 (S215) and (S235) are localized on the functional domain of these proteins, pointing to plausible phosphorylation-centric functional regulation. Further, we utilized the AlphaFold-predicted structural model of these proteins to map the spatial distribution of predominant phosphorylation sites [27]. We generated sequence coverage visualizations on the AlphaFold server using the following models: HNRNPM (AF-P52272-F1-v6), SLU7 (AF-O95391-F1-v6), average pLDDT 75.19, high confidence), and SRSF5 (AF-Q13243-F1). The AlphaFold model of SLU7 showed relatively high confidence; major sites S215 and S235 are located on different phosphopeptides within a flexible N-terminal loop, whereas S515 lies on a C-terminal α-helix. While SRSF5, S229, S231, and S233 form a clustered phospho-hotspot on the same flexible loop peptide, S250 is positioned on a separate peptide closer to the C-terminal. Predominant phosphorylation sites of HNRNPM occur on different structural compartments: S528, S618, and S633 appear in the flexible loop, while the S701 site is present on the α-helix within the RRM domain (Supplementary Figure S1). The localization of major phosphorylation sites outside the RBDs suggests significant allosteric regulation of RNA-binding function [11]. The structural localization of HNRNPM and SRSF5 phosphorylation sites with the low-confidence model should be considered for exploration and hypothesis generation only, as these predictions require experimental validation using approaches such as cross-linking mass spectrometry, NMR spectroscopy, cryo-EM, or molecular dynamics simulations before firm conclusions are drawn regarding their structural context or functional regulation.

### 2.3. Conserved Co-Differential Regulatory Patterns of Protein Phosphorylation with Phosphorylation of HNRNPM, SLU7, and SRSF5 at Their Predominant Sites

Based on cataloged global phosphoproteomic datasets and reproducible statistical analysis, positive/similar (UUDD) and negative/opposite (UDDU) co-differential phosphorylation of other proteins with phosphorylation of HNRNPM, SLU7 and SRSF5 at their predominant sites was derived (Supplementary Tables S7–S28). We identified a total of 66 negative and 584 positively co-differentially regulated protein phosphorylation sites with HNRNPM (S528) phosphorylation; 73 and 594 with HNRNPM (S618); 368 and 1489 with HNRNPM (S633); and 114 and 2675 with HNRNPM (S701). Similarly, 181 negative and 543 positively co-differentially regulated sites with SLU7 (S215), 96 and 171 with SLU7 (S515), and 966 and 2073 with SLU7 (S235) were identified. Also, 62 negative and 995 positively co-differentially regulated protein phosphorylation sites with SRSF5 (S233), 205 and 1032 with SRSF5 (S229), 362 and 1087 with SRSF5 (S250), and 323 and 448 with SRSF5 (S231) were identified. Further, we analyzed conservation of co-differential regulation events among the predominant phosphorylation sites of the same protein. Analysis showed that 57, 67, and 119 proteins were commonly co-differentially regulated with all the predominant phosphorylation sites of HNRNPM, SLU7, and SRSF5, respectively. A comparison of the top 10 high-confidence co-differential regulation events (based on their ratio of co-differential regulation) that positively and negatively co-differentially regulated with phosphorylation of HNRNPM, SLU7, and SRSF5 at predominant phosphorylation sites is represented in Figure 2.

### 2.4. Kinase–Substrate Interaction Network That Modulates mRNA Splicing Through Phosphorylation of RBDs

In order to seek major kinase families that potentially regulate mRNA splicing through RBD phosphorylation, we utilized network analysis to fetch high-confidence interactions among proteins that are commonly co-differentially phosphorylated with HNRNPM, SLU7, and SRSF5. Among the co-differentially regulated proteins, 811 proteins were commonly co-differentially regulated with the three phosphoproteins of interest, which were used to build a protein–protein interaction network using the STRING database (Figure 3A) [28]. Among the network, 24 proteins were found to contain functional domains belonging to the RBD superfamily (InterPro ID: IPR035979) (Supplementary Figure S2) while also being involved in splicing. Interestingly, network analysis displayed multiple proteins involved in mRNA splicing (Uniprot Keyword ID: KW-0508), but lacked RBDs, further confirming the importance of co-differential phosphoregulation analysis with implications in splicing. It was observed that in addition to certain serine-threonine kinases (Uniprot Keyword ID: KW-0723), multiple phosphoproteins involved in alternative splicing (which lacked RBDs) were also among commonly co-differentially regulated proteins (Uniprot Keyword ID: KW-0025). Most proteins in the network were involved in RNA splicing (Figure 3B), prompting us to investigate the synergy of phosphorylation in RNA binding and splicing.

Further, we mapped known phosphorylation sites on RBDs of 24 proteins involved in mRNA splicing, where several such sites showed conserved co-differential regulation with HNRNPM, SLU7, and SRSF5 phosphorylation (Supplementary Table S29). Kinases that potentially phosphorylate these sites were extracted to build a network (Supplementary Table S29) of experimentally known upstream kinases containing the RRM domain along with potential kinases derived through semi-experimental and predictive methods. It is noteworthy that several of the kinases potentially phosphorylating RBDs also showed co-differential regulation with HNRNPM, SLU7, and SRSF5 phosphorylation (Supplementary Table S30). To understand the major group of kinases that phosphorylate RBDs, we performed kinase enrichment analysis through the KinMap online tool, http://www.kinhub.org/kinmap/ (accessed on 15 October 2026) [29]. It was observed that Calcium/calmodulin-dependent protein kinases (CAMKs), followed by protein kinase A, G, and C families (AGC), majorly phosphorylated RBD-containing proteins involved in mRNA splicing, while tyrosine kinase-like (TKL) showed the least regulation (Figure 4A). Gene set enrichment analysis of the kinases revealed their involvement in MAPK, RTK, and cytokine signaling pathways, along with the enrichment of generic transcription pathways (Supplementary Table S31, Figure 4B). The analysis implies that kinases that potentially phosphorylate RBDs also regulate RNA binding through various signaling cascades.

## 3. Discussion

This study provides an in-depth analysis of the co-differential phosphorylation network of RBPs, through the lens of HNRNPM, SLU7, and SRSF5 phosphorylation. We systematically identified the predominant phosphorylation sites on these proteins and evaluated their functional relevance. Certain predominant phosphorylation sites of these proteins were located within functionally critical domains. For instance, the HNRNPM (S701) resides within the RRM domain essential for RNA binding, while S215 and S235 of SLU7 are localized within the Pre-mRNA Splicing Prp18-interacting factor domain. This domain is essential for the assembly of snRNAs (U1, U2, U4/U6, and U5) and associated proteins, catalyzing the excision of introns from pre-mRNAs via two successive transesterification reactions [30]. The localization of these phosphorylation sites within key RNA-binding and splicing domains suggests a significant impact on the functional regulation of these proteins, while extensive phosphorylation outside the functional domains pointed to allosteric regulations.

Additionally, we report a kinase regulatory network associated with commonly co-differentially regulated proteins, where 24 such phosphoproteins that contain RBDs are known to be involved in RNA recognition, splicing, transport, and post-transcriptional modifications [31]. Further analysis revealed 372 kinases responsible for phosphorylating these 24 phosphoproteins, highlighting extensive signaling interplay governing RNA processing and gene expression regulation. Additionally, investigation of conserved co-differential phosphorylation of protein phosphatases with HNRNPM, SLU7, and SRSF5 revealed that HNRNPM (S528, S618, S633, and S701) showed co-differential regulation with nine, 11, 24, and 42 phosphatases, respectively; SLU7 (S215, S515, and S235) showed co-differential regulation with eight, two, and 41 phosphatases, respectively; and SRSF5 (S233, S229, S250, and S231) showed co-differential regulation with 12, 10, 16, and nine phosphatases, respectively. Especially, RBD-specific phosphorylation will be of further interest in designing assays and therapies targeting conserved patterns. The results provide a framework for the phosphorylation-related regulation of RNA-binding activity, opening avenues for further experimental validation.

It is well established that mRNA splicing, alternative splicing and RNA binding are the three layers in the conversion of pre-mRNA to mature RNA transcripts [32]. Splicing provides the catalytic core that removes introns and ligates exons, alternative splicing introduces isoform diversification, and RNA-binding proteins supply the sequence-specific sensors that switch individual splicing events on or off [4,33]. In line with this framework, our study sought to construct a co-regulatory network that integrates these three aspects around the phospho-regulated splicing factors HNRNPM, SLU7, and SRSF5. The interaction network of commonly co-differentially regulated phosphoproteins comprised 16 proteins involved in alternative splicing, 54 proteins linked to core mRNA splicing, 28 identified as serine/threonine kinases, and 32 containing functional RBDs. This architecture highlights the functional significance of the co-regulating proteins uncovered in our analysis and suggests that systematic experimental interrogation of this network may reveal potential drug targets within the phosphorylation-controlled splicing pathway.

Further, an interaction network of commonly co-differentially regulated phosphoproteins was constructed by systematically mapping phosphorylation sites located within the RBDs of 14 proteins. In generating this network, all annotated phosphorylation sites within the RBDs of these 24 proteins were considered, rather than restricting the analysis solely to the subset of sites identified as co-differentially regulated in our dataset. Within this phosphorylation-centric network, several sites overlapped with our co-differential regulation data, highlighting specific points of convergence between global RBD phosphorylation and the observed co-differential regulatory patterns in this analysis. Notably, SRSF2 (S26) and SRSF6 (S45) co-differentially regulated with SRSF5 (S250), showing conserved co-differential regulation despite largely diverse experimental conditions and cell lines, indicating that this interesting co-differential regulation demands extensive exploration to functionally annotate the relationship. Interestingly, SRSF5 (S250) is localized on the RS domain, which is known to regulate protein–protein and protein–RNA interaction through phosphorylation [34]. As both SRSF2 (S26) and SRSF6 (S45) co-differentially regulated with SRSF5 (S250), this may signal co-functional regulation. A similar observation has been made with respect to SRSFs, where phosphorylation and dephosphorylation are involved in their nuclear transport and splicing [35]. Similarly, among the network of 372 upstream kinases that phosphorylate the 24 commonly co-differentially regulated, RBD-containing proteins, certain kinases exhibited coordinated co-differential regulation with specific, predominant phosphorylation sites on HNRNPM, SLU7, and SRSF5, revealing a robust kinase–substrate signaling network governing these splicing factors.

The mRNA splicing pathway is a crucial component of both physiological and pathological processes, involving the removal of introns and joining of exons through the spliceosome complex (U1, U2, U4, U5, U6 snRNPs) [36]. External stimuli activate signaling cascades that modulate splicing machinery activity and alter splicing patterns of target mRNA precursors, including Slo, NR1, CD44, CD45, and fibronectin genes [37]. The key regulatory mechanism involves signal-mediated changes in the phosphorylation state and subcellular distribution of splicing factors, particularly SR (serine/arginine-rich) proteins, which are essential trans-acting factors that bind to exons and intronic regions to regulate splice site selection [38]. Among the 113 upstream kinases phosphorylating the predominant sites of RBDs (HNRNPM, SLU7, and SRSF5), few kinases are directly involved in the mRNA splicing pathway. For example, SRPK1 and SRPK2 phosphorylate SR proteins in the cytoplasm to mediate their nuclear import, while CLK1, CLK3, and CLK4 phosphorylate SR proteins in the nucleus to regulate spliceosome assembly and alternative splicing patterns [39]. This phosphorylation/dephosphorylation cycle of SR proteins is critical for early steps of spliceosome assembly and transduces growth signals to regulate alternative splicing in the nucleus [40]. It makes these kinases essential mediators that connect extracellular signaling pathways to splicing regulation. We identified such specific kinase–substrate relationships in the co-differential regulation data, where SRPK2 (S497) acts as an upstream kinase of HNRNPM (S618); SRPK1 (S51, S311) is an upstream kinase of SLU7 (S235); CLK4 (S136, S138) phosphorylates HNRNPM (S618); and CLK1 (T138, S140), DCLK1 (S337), and CLK3 (S283, S157, S175, S173, S199) are upstream kinases of SLU7 (S235). STRING analysis further validated direct protein–protein interactions between SRPK2 (S497) and HNRNPM (S618). Interestingly, SRPK1 (S51, S311) interacted with SLU7 (S235), and SRSF5 interacted with DYRK1A, both of which are experimentally validated interactions in the STRING database, while the mechanism of action remains to be elucidated. This extensive co-differential regulation suggests that kinase signaling can coordinately tune multiple phosphorylation sites across key RNA-binding splicing factors, thereby providing a mechanistic route through which extracellular and intracellular cues can be translated into changes in spliceosome composition and alternative splicing outcomes.

Although this study successfully mapped the phosphorylation networks of key RBPs to offer significant insights into their roles in RNA metabolism, splicing, and apoptotic regulation, certain limitations persist and provide prospects. While our findings lay a robust foundation for understanding the phosphorylation-dependent regulation of HNRNPM, SLU7, and SRSF5 through both RBD-specific and allosteric regulation, future experimental validation, including structural studies and functional assays, is essential. Although multiple inclusion and exclusion criteria were employed to select high-confidence co-differentially regulated protein phosphorylation sites, the lack of experimental validation restricts our ability to verify their direct functional relevance. While key parameters affecting detection sensitivity, including quantification methods, mass spectrometry platforms, and enrichment protocols, were incorporated into our stratification criteria, greater resolution could be attained by systematically grouping datasets according to additional experimental variables. The analysis integrates publicly available phosphoproteomic datasets generated under diverse experimental conditions, using different sample types, cell lines, biological perturbations, sample-preparation procedures, and phosphopeptide enrichment strategies. Although stringent primary and secondary filtering criteria were applied to identify high-confidence phosphorylation patterns, residual technical and biological heterogeneity cannot be completely excluded, particularly because the datasets were not generated within a single standardized experimental pipeline. The co-regulatory associations identified in this study may be influenced by unequal representation of datasets, correlated measurements across studies, differences in phosphorylation site detectability, and variation in protein abundance. Consequently, the observed patterns reflect statistical co-regulation rather than direct causal kinase–substrate relationships. The predicted regulatory associations involving HNRNPM, SLU7, and SRSF5 phosphorylation have not yet been completely experimentally validated. Given the large number of phosphorylation sites, candidate kinases, interacting proteins, and heterogeneous biological contexts included in this analysis, comprehensive wet-laboratory validation was not feasible at the present stage. Therefore, the findings should be interpreted as hypothesis-generating rather than as definitive evidence of regulatory mechanism. In addition, phosphorylation sites identified primarily through mutation-based approaches or phosphorylation site-specific antibodies but not frequently detected or classified as Class I in discovery-based phosphoproteomic datasets were not included in the current analysis. Such targeted studies typically focus on predefined sites and do not provide an unbiased, proteome-wide assessment of phosphorylation. In contrast, this study leverages discovery-based mass spectrometry datasets that survey phosphorylation across entire proteins, thereby enabling systematic ranking of detectable RBD phosphorylation based on recurrence and co-modulation patterns. Future studies should prioritize the highest-confidence HNRNPM, SLU7, and SRSF5 phosphorylation sites and their candidate regulatory partners for targeted experimental validation.

## 4. Methodology

### 4.1. Assessment and Congregation of Global Phosphoproteomic Datasets

The phosphorylation landscape and co-differential regulation pattern of protein phosphorylation of HNRNPM, SLU7, and SRSF5 was cataloged by systematically screening PubMed using the search terms “phosphoproteomics” OR “phosphoproteome” NOT “Plant” NOT “Review”. High-throughput phosphoproteomic datasets derived from human cell lines were assembled to integrate quantification of phosphopeptides derived from proteolytic cleavage of phosphoproteins by LysC and/or trypsin enzymes. These datasets were also classified based on phosphopeptide enrichment techniques (STY/ST/Y phosphorylation sites). To compile high-confidence data, only Class 1 phosphorylation sites (localization probability ≥ 75% or ambiguity score > 13) were considered for further classification as profile and differential datasets. The profile data includes the phosphorylation site-derived phosphoproteomic profiling datasets that showed overall quantities of phosphopeptides in all samples. The datasets that provided comparative quantification of specific biological or experimental conditions to their respective control were categorized as differential, where phosphopeptides with the absolute fold change ≥ 1.3 are considered to be upregulated, while those with absolute fold change ≤ 0.76 are considered to be downregulated (*p*-value < 0.05). To ensure uniform annotation post-data cataloging, each protein was mapped to its corresponding HGNC gene symbol, and each phosphorylation site was linked to its UniProt accession using in-house mapping tools [41,42]. Additionally, all biological and experimental conditions were standardized and tagged according to established formats [43,44,45].

### 4.2. Identification of Major Phosphorylation Hotspots of HNRNPM, SLU7, and SRSF5

Cataloged phosphorylation sites were ranked according to the number of times they were identified in profile and differential datasets (frequency) in various biological experimental conditions. Major phosphorylation hotspots of HNRNPM, SLU7, and SRSF5 were identified by this ranking. The phosphorylation sites detected in over 50% of the profile and differential datasets were designated as major phosphorylation sites or predominant phosphorylation sites of HNRNPM, SLU7, and SRSF5.

### 4.3. Integrated Statistical Analysis for Identifying High-Confidence Protein Phosphorylation Sites That Showed Conserved Co-Differential Regulation with Predominant Phosphorylation Sites of HNRNPM, SLU7, and SRSF5

The phosphorylation patterns of other proteins, which showed positive (similar) or negative (opposite) co-differential regulation with phosphorylation of HNRNPM, SLU7, and SRSF5, were identified by analyzing the differential datasets [46,47]. Data was categorized into upregulation and downregulation according to the differential expression of phosphopeptides containing predominant phosphorylation sites. From these datasets, protein phosphorylation events that are upregulated (U) and downregulated (D) with the predominant phosphorylation sites upregulated (UU and UD) and downregulated (DU and DD) were extracted. Subsequently, UU and DD events were categorized as positive co-differential regulation and UD and DU events were categorized as negative co-differential regulation with phosphorylation of HNRNPM, SLU7, and SRSF5. This resulted in a phosphorylation site-specific co-differential regulation list (list of positive and negative co-differentially regulated phosphorylation events for each predominant site), which was used for further statistical analysis.

In order to fetch conserved co-differential regulatory patterns among positive (UUDD) and negative (UDDU) co-differential regulation events and to avoid the false-positive results/biasing, we performed one-sided Fisher’s exact test (FET). Analysis was conducted for a pair of phosphorylation sites (a predominant site as compared to a phosphorylation site on a co-differentially regulating protein). The contingency table used to calculate the FET *p*-value included four variables that accounted for the number of differential datasets in which (i) both site A and site B were differentially regulated; (ii) site A was differentially regulated, whereas site B was not; (iii) site B was differentially regulated, whereas site A was not; (iv) neither site A nor site B was differentially regulated. Because each RBD phosphorylation site was tested against a large number of phosphorylation sites, the resulting *p*-values were corrected for multiple hypothesis testing using the Benjamini–Hochberg false discovery rate (BH-FDR) procedure. The resulting q-values represent the minimum false discovery rate at which each individual test would be considered significant. This correction was applied across the complete set of phosphorylation site pair combinations evaluated for the predominant HNRNPM, SLU7, and SRSF5 phosphorylation sites. Site pair associations were considered statistically significant at q < 0.05. To quantify the magnitude of co-differential regulation, effect sizes were additionally calculated as odds ratios with 95% confidence intervals.

The phosphorylation site pairs were chosen to show conserved co-differential regulation when their FET *p*-value was <0.05, FDR q-Value < 0.05 and odds ratio > 2. Further, high-confidence co-differential regulation phosphorylation sites were filtered from the FET results by implementing secondary data filtering criteria based on the ratio of Ʃ(nUU + nDD)/Ʃ(nUD + nDU) for positive co-differential regulation, and Ʃ(nUD + nDU)/Ʃ(nUU + nDD) for negative co-differential regulation. The protein phosphorylation site pair that obeys the ratio of ≥10% of the total frequency of the predominant phosphorylation sites was considered to be significant and was selected for further analysis. Such co-differential regulatory events were only picked when co-differential regulation was observed in 3 distinct studies and in at least three differential experimental conditions (represented by experimental code). Protein phosphorylation site pairs that satisfied these four criteria and displayed either positive or negative co-differential regulation were regarded as high-confidence and conserved.

### 4.4. Identification of Known and Predicted Upstream Kinases and Binary Interactors of HNRNPM, SLU7, and SRSF5

Next, we collated upstream kinases and binary interactors of HNRNPM, SLU7, and SRSF5. Experimentally validated upstream kinases that phosphorylate the predominant phosphorylation sites on these proteins were extracted from databases including PhosphoSitePlus, http://www.phosphosite.org (accessed on 22 May 2023) [48], Phospho.ELM 9.0, http://phospho.elm.eu.org (accessed on 24 May 2023) [49] and RegPhos 2.0, http://csb.cse.yzu.edu.tw/RegPhos2/ (accessed on 24 May 2023) [50]. Similarly, predicted upstream kinases were obtained from NetworKIN, http://networkin.info (accessed on 4 January 2023) [51], Automatic Kinase-specific Interaction Detection, (AKID) http://akid.bio.uniroma2.i (accessed on 24 May 2023) [52], and a high-throughput method based on an in vitro screen of kinases as provided in iKiP-DB [53]. Additionally, potential upstream kinases were also derived from synthetic peptide library screening assays by Johnson et al. (2024) (90% cutoff) [54]. Additionally, kinases and phosphatases were annotated from the FET analysis by utilizing protein functional ontology mapping [55]. Similarly, experimentally validated protein–protein interactions were retrieved from databases including BioGRID version 5.0, http://thebiogrid.org/ [56], ConsensusPathDb (version 35) http://consensuspathdb.org (accessed on 22 May 2023) [57], CORUM comprehensive resource of mammalian protein complexes, https://mips.helmholtz-muenchen.de/corum/ (downloaded 1 March 2023) [58] and RegPhos (version 2.0), http://csb.cse.yzu.edu.tw/RegPhos2/ (downloaded 1 May 2023) [50].

### 4.5. Kinase-Mediated Phosphorylation Network of RBPs Involved in Splicing

Investigation of kinase regulatory networks associated with RBPs involved in splicing regulation was performed by focusing on three RBPs, HNRNPM, SLU7, and SRSF5, owing to their well-documented roles in RNA processing and shared functional characteristics. Initially, a list of commonly co-differentially regulated proteins that showed a significantly conserved co-differential phosphorylation pattern (FET *p*-value < 0.05) with all 3 proteins (HNRNPM, SLU7, and SRSF5) was extracted. Next, the STRING database (version 12.0) [59] was used to extract interactions between these common proteins (confidence = 0.7, with associations from experimental, database, and co-expression sources). To further refine our network, we fetched the proteins involved in splicing (containing RBDs) and serine/threonine kinases to catalog corresponding biological function enrichment results from STRING network analysis. The proteins containing any domain belonging to the RBD superfamily and involved in splicing were extracted to map co-differentially regulated phosphorylation sites of these proteins which were localized on the corresponding RBD. Further, upstream kinases responsible for phosphorylation at these sites were retrieved by the same methodology used above (Section 4.4). Finally, using the curated list of phosphorylation sites and their upstream kinases, we constructed a kinase regulatory network representing the dynamic phosphorylation-mediated regulation of RBPs in splicing. To investigate the functional involvement of upstream kinases phosphorylating RBPs, gene set enrichment analysis was performed through g:Profiler, https://biit.cs.ut.ee/gprofiler (downloaded on 24 October 2026) [60].

### 4.6. Visualization

The visualization of phosphorylation sites in profile and differential datasets of HNRNPM, SLU7 and SRSF5 is represented in a lollipop plot obtained using the TrackViewer R package (10.18129/B9.bioc.trackViewer). Additionally, circular dendrograms were constructed using RAWGraph version 2.0 [61]. The kinase regulatory network was built using Cytoscape version 3.10.3 [62]. Kinase family enrichment was performed using the KinMap tool, http://www.kinhub.org/kinmap/ [29]. Bar charts were generated using R packages including dplyr version 1.2.1 [63] (for data preparation), ggplot2 version 4.0.3 [64], pheatmap version 1.0.13 [65], and tidyr version 1.3.2 [63] (for data preparation).

## 5. Conclusions

In this study, we attempted to comprehensively explore the phosphorylation network of the RBPs, specifically HNRNPM, SLU7, and SRSF5, along with their co-differentially regulated protein phosphorylation sites. We identified key phosphorylation sites within these proteins, with particular emphasis on those located within functionally significant domains. Furthermore, we constructed a kinase regulatory network associated with co-differentially regulated phosphorylation sites of proteins harboring RBDs. This network offers valuable insights into the complex signaling pathways influencing RBP function and provides a foundation for therapeutic investigations targeting phosphorylation-mediated regulation. Despite this extensive network analysis, critical knowledge gaps remain: (1) the precise mechanistic relationships between specific phosphorylation events and downstream functional consequences on RBP activity, substrate specificity, and cellular outcomes are not fully elucidated, and (2) the temporal dynamics and context-dependency of these phosphorylation events across different cell types are not fully understood, requiring developmental integrated multi-omics approaches combining phosphoproteomics with transcriptomics and single-cell resolution analyses. Further biochemical validation through in vitro kinase assays, co-immunoprecipitation, and mutagenesis studies are essential to confirm predicted interactions. Further functional and structural studies, including crystallography and cryo-EM analyses of phosphorylated RBP complexes, will be crucial in translating these findings into clinically relevant applications and developing targeted therapeutic interventions for diseases involving dysregulated RBP phosphorylation. Overall, this framework establishes a platform for future in-depth studies to delineate the precise roles of HNRNPM, SLU7, and SRSF5 in cellular signaling, their interaction networks, and their potential as diagnostic targets in disease contexts.

## Figures and Tables

**Figure 1:**
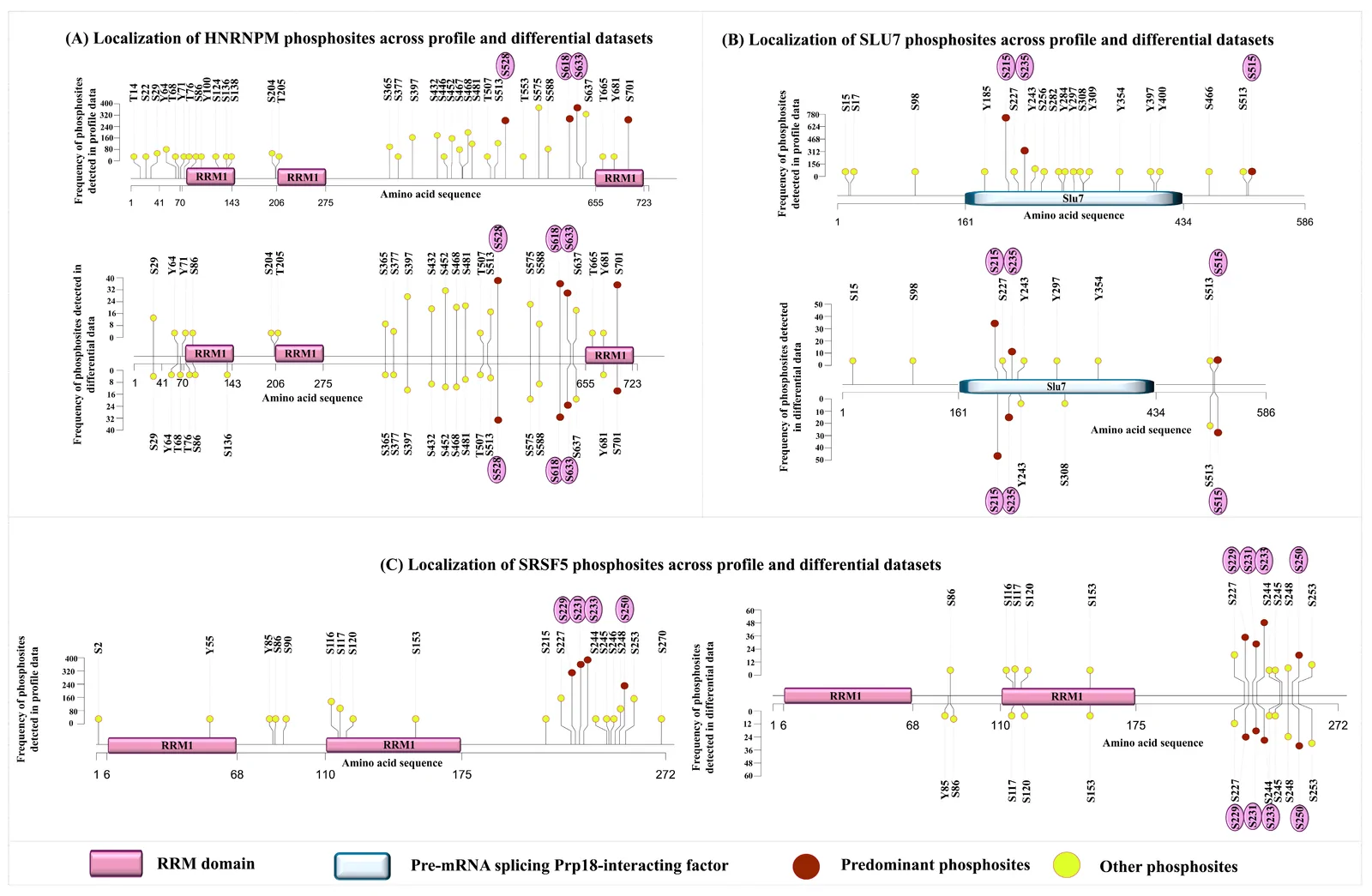
Lollipop plot visualization of phosphorylation sites of HNRNPM, SLU7 and SRSF5 with the frequency of detection in global cellular phosphoproteomics. (**A**) Phosphorylation sites detected across the profile and differential datasets of HNRNPM; (**B**) phosphorylation sites detected across the profile and differential datasets of SLU7; (**C**) phosphorylation sites detected across the profile and differential datasets of SRSF5.

**Figure 2:**
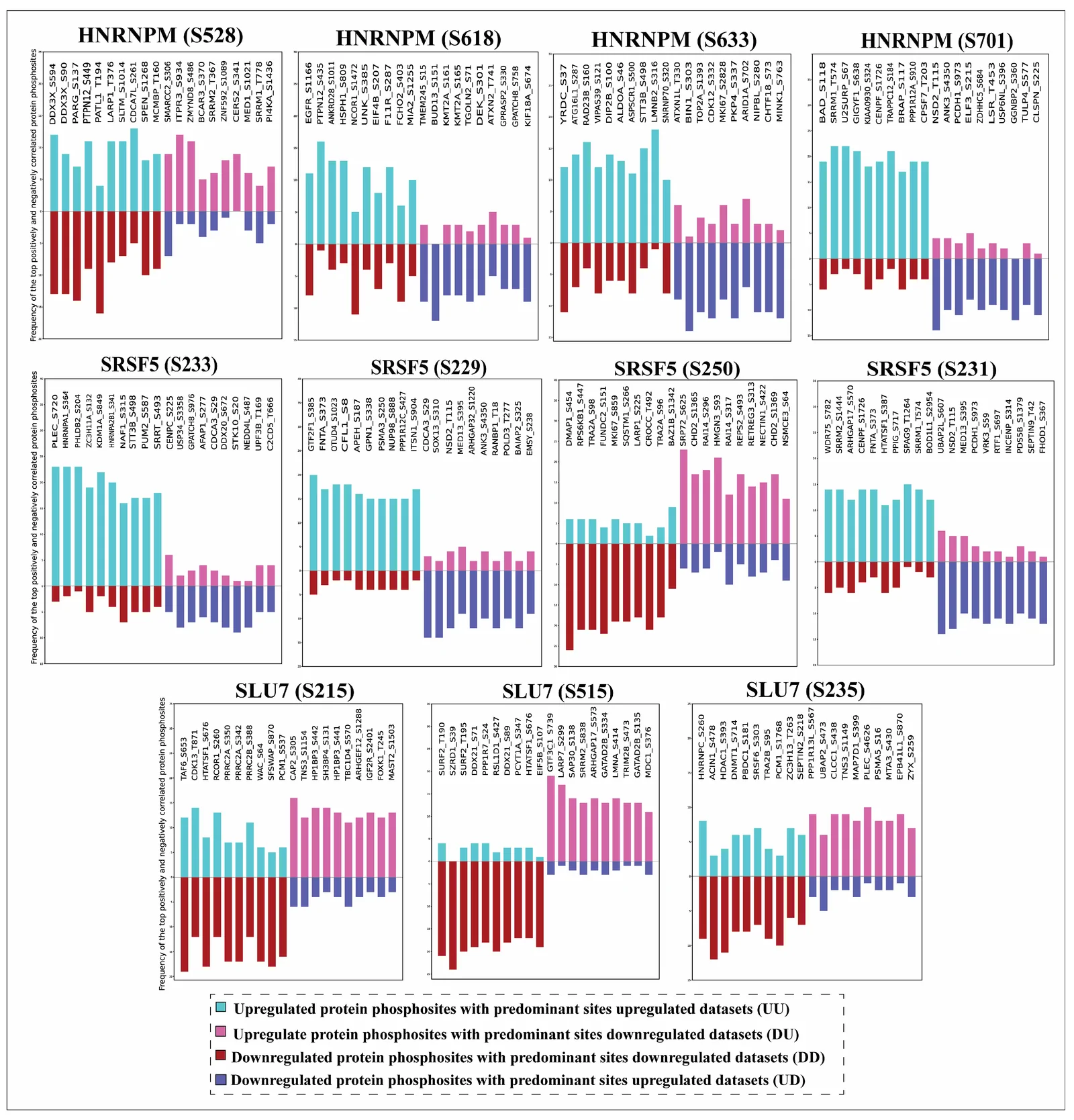
Bar plot representing the top 10 co-differentially regulated protein phosphorylation sites that are positively/negatively co-differentially regulated with the phosphorylation at predominant sites of HNRNPM, SRSF5, and SLU7.

**Figure 3:**
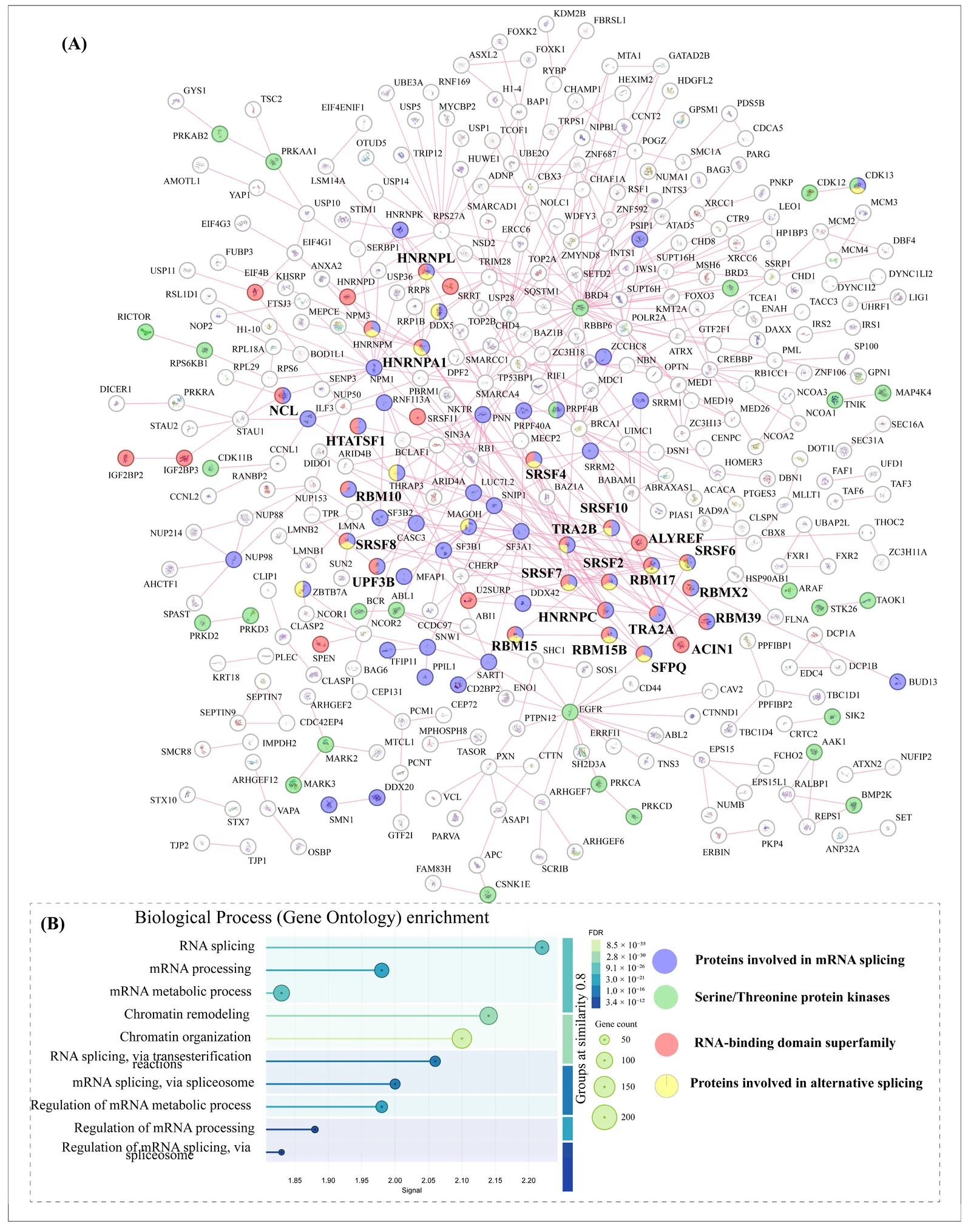
Interaction network of commonly co-differentially regulated phosphoproteins with HNRNPM, SLU7, and SRSF5 (generated through the STRING database). (**A**) Interaction network highlighted with proteins containing RBDs involved in splicing; (**B**) biological processes enriched for commonly co-differentially regulated phosphoproteins.

**Figure 4:**
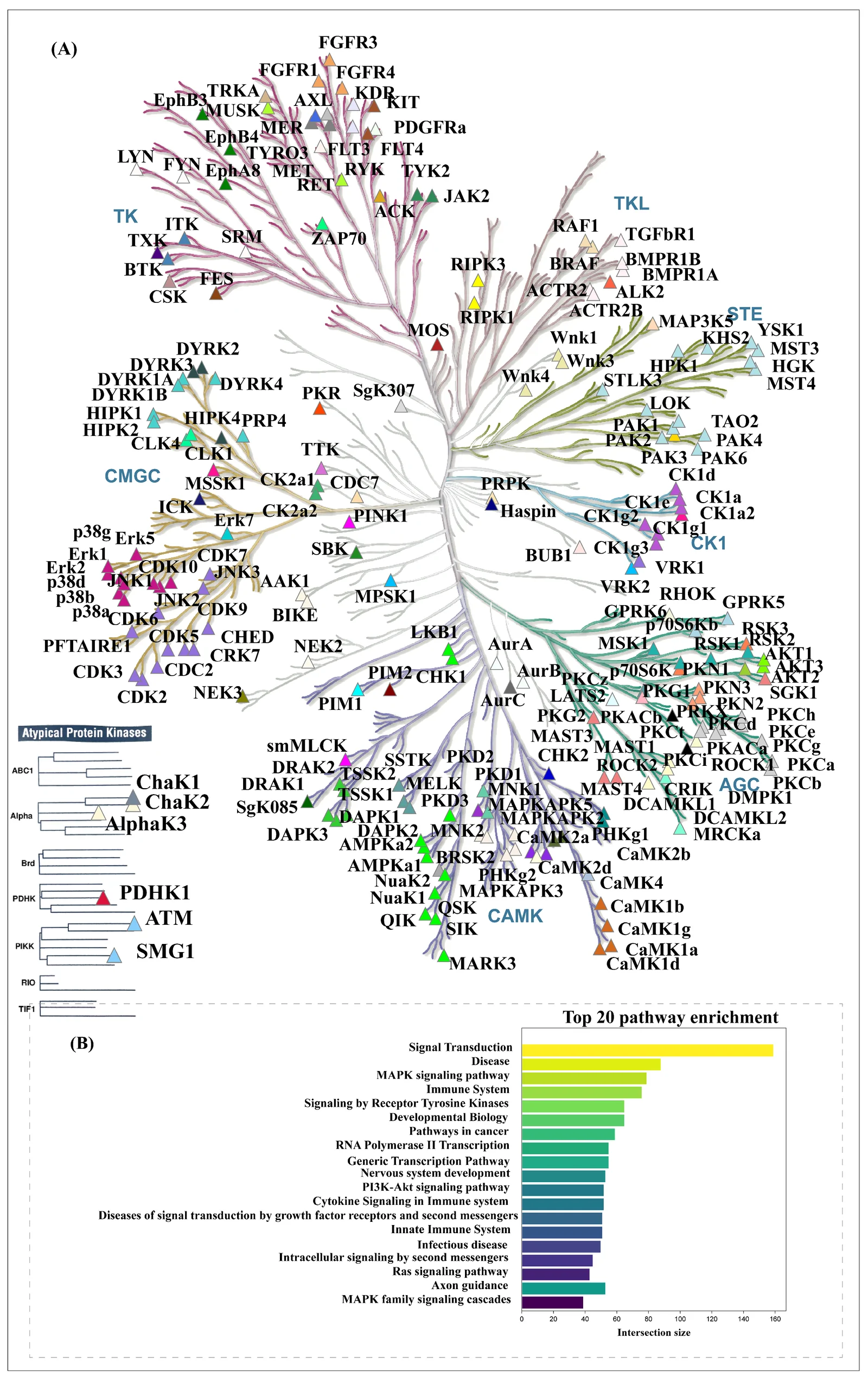
Upstream kinases modulating RBD phosphorylation in splicing. (**A**) The figure illustrates 372 kinases that are potential upstream kinases of co-differentially regulated protein phosphorylation sites containing RBDs and involved in splicing. The figure was generated through the KinMap tool. (**B**) Pathway enrichment results illustrate significantly enriched pathways that are modulated by the upstream kinases.

## Data Availability

The original contributions presented in this study are included in the article/Supplementary Materials. Further inquiries can be directed to the corresponding authors.

## Supplementary Materials

The following supporting information can be downloaded at https://www.mdpi.com/article/10.3390/ijms27219965/s1.
