# Supplementary material for: RNA-Binding Proteins in mRNA Splicing: Orchestration of Altered Phosphorylation of RNA-Binding Domains via Kinase Regulatory Networks
Source: Int J Mol Sci. 2026 Nov 6;27(21):9965. doi: 10.3390/ijms27219965 (PMC13764065; doi:10.3390/ijms27219965)
Supplement: Supplementary file 1 [file ijms-27-09965-s001.zip › Supplementary figures.pdf]

RNA-binding proteins in mRNA splicing: Orchestration of altered phosphorylation of RNA-binding Domains via kinase regulatory networks

Ashika Bangera[1], Samseera Ummar[1], Alimath Sambreena[1], Athira Perunelly Gopalakrishnan[1], Poornima Ramesh[1], Rajesh Raju[1]**

[1]Centre for Integrative Omics Data Science (CIODS), Yenepoya (Deemed to be University), Mangalore 575018, India

Correspondence should be addressed to

Rajesh Raju, Ph.D
Professor
Centre for Integrative Omics Data Science,
Yenepoya (Deemed to be University),
Mangalore, Karnataka- 575018, India
Email: rajrrnbt@gmail.com; rajeshraju@yenepoya.edu.in

And

Poornima Ramesh, Ph.D
Assistant Professor,
Centre for Integrative Omics Data Science,
Yenepoya (Deemed to be University),
Mangalore, Karnataka, 575018, India
Email: poornima.devadhar@gmail.com; poornima_r.ciods@yenepoya.edu.in

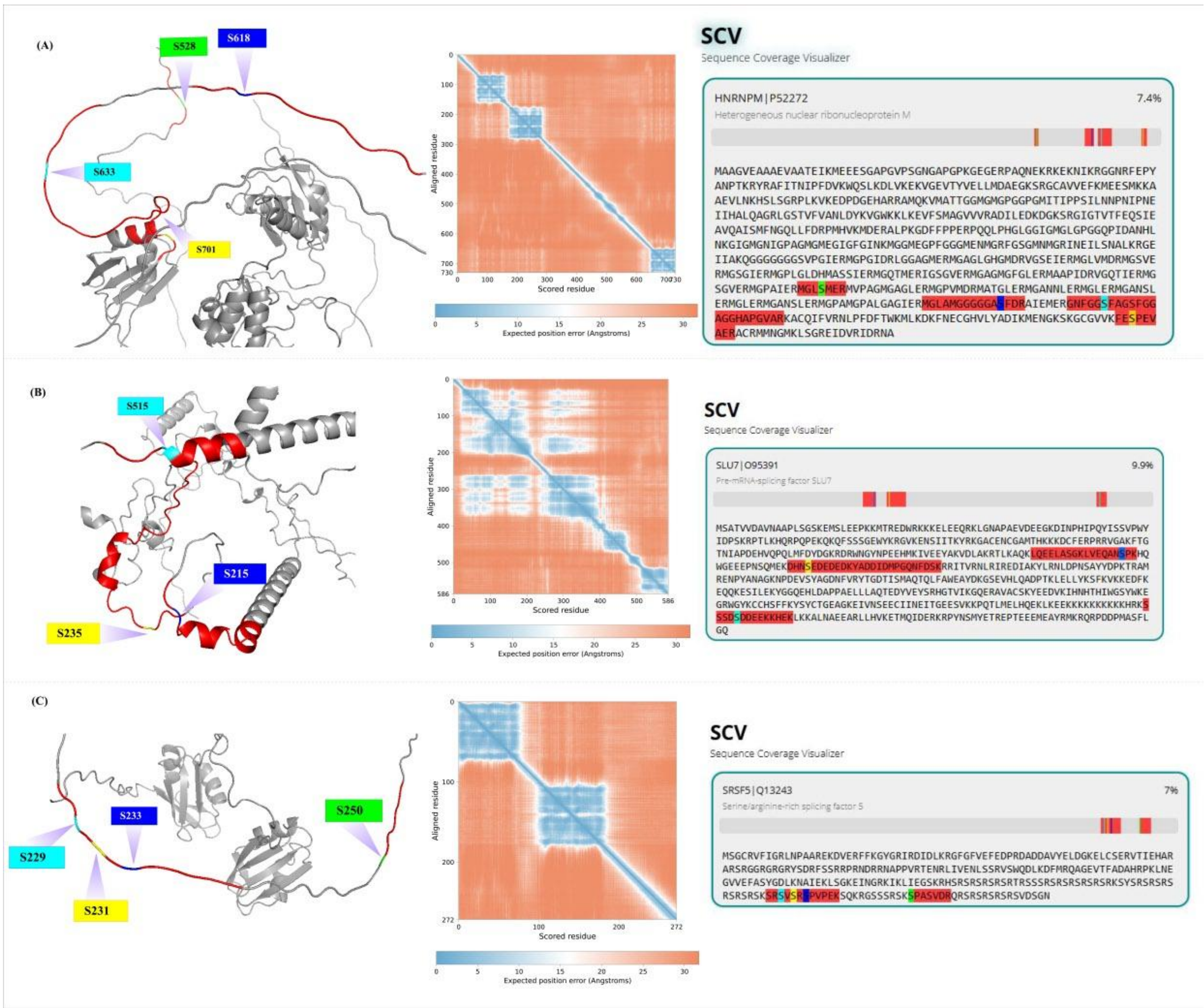

Supplementary figure S1: AlphaFold predicted structural models of HNRNPM, SLU7, and SRSF5 with mapped spatial distribution of predominant phosphorylation sites. **(A)** Mapped predominant phosphorylation sites of HNRNPM into the AlphaFold model with their sequence motif; **(B)** mapped predominant phosphorylation sites of SLU7 into the AlphaFold model with their sequence motif; **(C)** mapped predominant phosphorylation sites of SRSF5 into the AlphaFold model with their sequence motif.

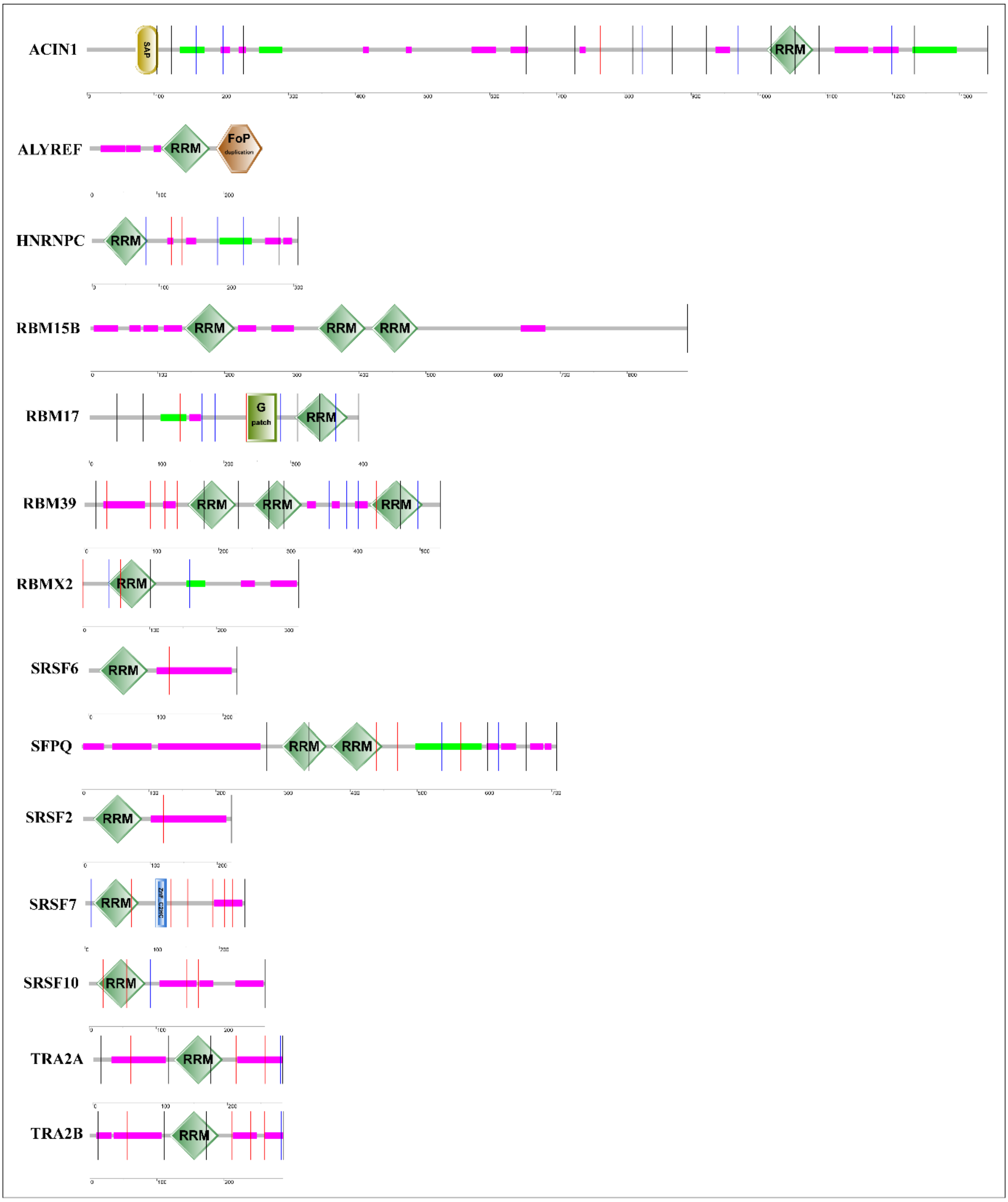

Supplementary figure S2: Functional domain of 14 proteins belonging to the RBD superfamily.
